# Supplementary material for: COZOID: contact zone identifier for visual analysis of protein-protein interactions
Source: BMC Bioinformatics. 2018 Apr 6;19:125. doi: 10.1186/s12859-018-2113-6 (PMC5889581; doi:10.1186/s12859-018-2113-6)
Supplement: Supplementary file 2 — Software build. Executable binary file of the software tool. (ZIP 118,784 kb) [file 12859_2018_2113_MOESM2_ESM.zip › cozoid/java/docs/beansbinding-1.2.1-doc.zip!/org/jdesktop/swingbinding/adapters/package-frame.html]

org.jdesktop.swingbinding.adapters (Beans Binding)


org.jdesktop.swingbinding.adapters

Classes 
  
AbstractButtonAdapterProvider
  
AbstractButtonAdapterProvider.Adapter
  
BeanAdapterBase
  
JComboBoxAdapterProvider
  
JComboBoxAdapterProvider.Adapter
  
JListAdapterProvider
  
JSliderAdapterProvider
  
JSliderAdapterProvider.Adapter
  
JSpinnerAdapterProvider
  
JSpinnerAdapterProvider.Adapter
  
JTableAdapterProvider
  
JTextComponentAdapterProvider
